# Recognizing and Grading CAR T-Cell Toxicities: An Advanced Practitioner Perspective

**DOI:** 10.3389/fonc.2020.00885

**Published:** 2020-06-24

**Authors:** Savannah Sievers, Grace Watson, Swapna Johncy, Sherry Adkins

**Affiliations:** ^1^Department of Physician Assistant Programs, The University of Texas MD Anderson Cancer Center, Houston, TX, United States; ^2^Department of Lymphoma and Myeloma, The University of Texas MD Anderson Cancer Center, Houston, TX, United States

**Keywords:** CAR T-cell, advanced practice providers, cytokine release syndrome, immune effector cell-associated neurotoxicity, differential diagnosis

## Abstract

Over the past decade, chimeric antigen receptor (CAR) T-cell therapy has significantly improved the outlook for many patients with relapsed and/or refractory B-cell malignancies. The use of CAR T-cell therapy and other therapeutic immune effector cells will likely continue to expand with the development of other targets and use in solid tumors. Although these therapies have shown significant promise in the treatment of some malignancies, they can be associated with unique toxicities including cytokine release syndrome and immune effector cell-associated neurotoxicity syndrome which can be fatal if not identified early and treated appropriately. An understanding of how best to manage the toxicities associated with CAR T-cell therapy is continually evolving. Institutions providing CAR T-cell therapy have undergone changes in infrastructure and staffing models in order to safely care for patients receiving this novel therapy. As members of a multi-disciplinary health care team, advanced practice providers play significant roles in caring for this patient population and must be well-versed in the recognition, grading, and appropriate management of CAR T-cell therapy-related toxicities as these providers care for patients in multiple settings across the continuum of care.

## Introduction

Chimeric Antigen Receptor (CAR) T-cell therapy is an adoptive cell therapy that has dramatically improved outcomes in some patients with relapsed or refractory B-cell malignancies. To date, the US Food and Drug Administration (FDA) has approved two constructs that target B-cell malignancies expressing CD19 on the cell surface, and there are numerous clinical trials for agents targeting other antigens for both hematologic malignancies and solid tumors. Third- and fourth-generation CAR T-cell therapies, including allogeneic products, are also undergoing clinical investigations (1–8). As more cell products become commercially available and more authorized treatment centers are approved, access to this highly specialized treatment will continue to increase (9, 10).

Despite promising results of clinical trials, toxicities associated with CAR T-cell therapy are common and can be fatal. Although the mechanisms driving toxicities are becoming better understood, the long term sequelae have yet to be determined (11–14). Therefore, it is vital that high level multidisciplinary teams are in place to correctly identify, grade, and manage these adverse events (15). Advanced practice providers (APPs) in the United States experience regular patient contact, and are often responsible for the initial assessment of patients who have received CAR T-cell therapy. Therefore, they must be knowledgeable of the expected toxicities, the timing and grading of these toxicities, and the signs and symptoms of other adverse events that may accompany or mimic the toxicities associated with CAR T-cell therapy (16). Although consensus guidelines on the grading of CAR-T cell-related toxicities were recently published, challenges remain in accurately implementing these guidelines (17, 18).

The purpose of this focused review is to highlight the influence of multidisciplinary care teams, including APPs, in correctly identifying and grading CAR T-cell-related toxicities. This review will include a focused discussion regarding risk factors, differential diagnoses, and guidelines for grading cytokine release syndrome (CRS) and immune effector cell-associated neurotoxicity syndrome (ICANS), as well as long-term and late effects of CAR T-cell therapy and ways APPs contribute to providing optimal care.

## Cytokine Release Syndrome (CRS)

Of the reported toxicities related to CAR T-cell therapy, CRS is the most common, and without timely intervention, it can be fatal. CRS is defined by the American Society for Transplantation and Cellular Therapy (ASTCT) consensus group as the “supra-physiologic response following immune therapy that results in activation or engagement of endogenous or infused T cells.” According to the ASTCT, CRS begins with a fever and can include hypotension, hypoxia, and/or end organ dysfunction, and these symptoms may be progressive (17). Other adverse events involved in CRS may include arrhythmias, cardiomyopathy, heart block, renal failure, transaminitis, and coagulopathy. However, the hallmark of CRS is fever, which is the first presenting symptom, and the severity of CRS is determined by hypotension and/or hypoxia (17). CAR T-cell experts have concluded that the other significant adverse events of CRS are uncommon in the absence of hypotension and/or hypoxia. CRS typically occurs within the first 1 to 14 days following CAR T-cell infusion and can last 2–3 weeks; although this often resolves sooner with optimal management (19, 20). Still, it is recommended that patients be closely monitored for CRS for at least 4 weeks after CAR T-cell infusion (10).

### Grading for CRS

In the past, grading for CRS has varied across institutions (12, 21–23). While each grading system improved recognition of CRS, there remained a lack of consensual grading that would allow for comparison among trials and products. To remedy this, experts in CAR T-cell therapy met in 2018 to formalize official recommendations for grading CAR T-cell related toxicities. The final consensus from the ASTCT can be found in Table 1, and is the system most widely adopted by institutions within the United States.

**Table 1:** American Society for transplantation and cellular therapy consensus grading of cytokine release syndrome (CRS).

CRS parameter^*^	**Grade 1**	**Grade 2**	**Grade 3**	**Grade 4**
**Fever**^**#†**^	Temperature ≥ 38°C	Temperature ≥ 38°C	Temperature ≥ 38°C	Temperature ≥ 38°C
		**With either:**		
**Hypotension**^**#**^	None	Not requiring vasopressors	Requiring one vasopressor with or without vasopressin	Requiring multiple vasopressors (excluding vasopressin)
		**And/or**^**‡**^		
**Hypoxia**^**#**^	None	Requiring low-flow nasal cannula^∧^ or blow-by	Requiring low-flow nasal cannula^∧^, facemask, non-rebreather mask, or Venturi mask	Requiring positive pressure (e.g., CPAP, BiPAP^**^, intubation and mechanical ventilation

* *Organ toxicities associated with CRS may be graded according to Common Terminology for Adverse Events version 5.0, but these toxicities do not influence CRS grading*.

# *Not attributable to any other cause*.

*In patients who have CRS and then receive tocilizumab or steroids, fever is no longer required to grade subsequent CRS severity*.

‡ *CRS grade is determined by the more severe event*.

∧ *Low-flow nasal cannula is ≤ 6 L/min and high-flow nasal cannula is >6 L/min*.

** *CPAP, continuous positive airway pressure; BiPAP, bilevel positive airway pressure*.

*Table adapted from (17)*.

While a history of the grading of CRS is important, because many clinical trials still utilize past grading systems, the aim of this review is focused on highlighting common errors that can meaningfully affect toxicity reporting and grading. For instance, elevated liver enzymes weeks after CAR T-cell infusion are less likely attributable CAR T-cells than to a secondary cause such as antifungal therapy. Additionally, while labs such as C-reactive protein and ferritin levels can help aide in diagnosis, they can also be elevated in numerous other conditions. The next sections will address risk factors and differential diagnoses of CRS to help determine whether a symptom, lab abnormality and adverse event is attributable to CAR T-cell therapy.

### Risk Factors for CRS

CRS incidence is high and varies on the basis of a number of factors. In fact, the focus of many clinical trials has been to identify potentially modifiable risk factors because understanding these risk factors can aide in prompt diagnosis (19). High disease burden is one of the most important predictors of severe CRS (24, 25). This has been validated in studies of patients with acute lymphoblastic leukemia who received CAR T-cell therapy as well as in lymphoma models (26–28). Additionally, high T-cell doses and a high degree of T-cell expansion can increase the risk of developing CRS. Early cytokine elevations have been associated with severe CRS, and have the potential to act as predictive biomarkers for the development of severe CRS (25, 27). Furthermore, the incidence of CRS varies with the type of CAR T-cell agent and construct, including among the second-generation FDA-approved CAR T-cell products. However, owing to differences in patient populations and grading scales, no definitive conclusions can be drawn.

### Differential Diagnoses for CRS

The lymphodepleting chemotherapies most commonly used prior to CAR T-cell infusion- cyclophosphamide and fludarabine- are known to cause cytopenias, which occur during the same timeframe that is typical for CRS. The CAR T-cells themselves may also cause immune-mediated pancytopenia. Thus, it is often difficult to determine whether patients with a fever and neutropenia are experiencing CRS or an infectious complication. One multidisciplinary team noted that infection is more likely to occur in patients who receive high CAR T-cell doses, are heavily pre-treated, or experience severe CRS (29). In fact, the most predictive risk factor for infection in that report was CRS severity. In patients who have received CAR T-cell therapy and subsequently develop fever, an infectious work-up is indicated and empiric antimicrobials should be given until infection is ruled out, especially in patients with neutropenia.

Sepsis presents similarly to CRS, and it is often extremely challenging to distinguish between the two. To illustrate, a substantial number of patients with CRS also meet the criteria for sepsis, which is defined as suspected infection with organ dysfunction (30, 31). Furthermore, elevated lactate levels and hypotension that requires vasopressor support meet the criteria for septic shock and may also occur in patients with severe CRS (24). Although the supportive care for both adverse events may be similar, it is imperative to determine the underlying cause of the symptoms—CRS vs. infection—because the treatment varies drastically. Treatment for CRS may include anti-cytokine therapy and high-dose steroids, which would not be indicated in sepsis. Therefore, it is of value to consider sepsis as a likely differential diagnosis when managing symptoms in a patient who has received CAR T-cell therapy.

Tumor lysis syndrome (TLS) can also present similarly to CRS. Clinically, the symptoms of tumor lysis syndrome may include fever, renal failure, arrhythmia, and seizures. However, tumor lysis syndrome can be identified using objective data such as abnormal laboratory findings including hyperphosphatemia, hyperuricemia, hypocalcemia, and hyperkalemia (24).

Although rare, hypersensitivity reactions to CAR T-cell therapy can occur, and a few cases have been presented in the literature (32). Hypersensitivity presents most often after repeat exposure, which, again, is rare for patients receiving CAR T-cell therapy. The presentation of a typical type I hypersensitivity reaction includes fever, urticarial rash, hypotension, dyspnea, and gastrointestinal symptoms, all of which can been seen in CRS. However, hypersensitivity reactions occur at the same time or soon after the cell infusion, which is not typical for CRS.

In the context of differential diagnoses for CRS, hemophagocytic lymphohistiocytosis (HLH), also termed macrophage activation syndrome, is a rare but potentially fatal condition that can occur in conjunction with CRS (24). As with the previous differential diagnoses, HLH symptoms overlap with those of CRS. To diagnosis HLH, a combination of clinical, laboratory, and histopathological findings must be present (33). According to the most recent guidelines from the Histiocyte Society, diagnosis requires molecular mutations (i.e., *PRF1, UNC13D, STXBP1*, etc.) or five of the following criteria: fever, splenomegaly, cytopenia affecting at least two different cell lines, hypertriglyceridemia, hemagophagocytosis in the bone marrow, spleen, lymph nodes or liver; low or absent natural killer cells, elevated ferritin and increased soluble CD25 concentration (i.e., soluble IL-2 receptor), the latter of which also fits the clinical picture of CRS (34). However, while HLH is rare in patients receiving CAR T-cells, CRS is common, so CRS should remain high on the differential.

Other important differential diagnoses for CRS include heart failure, pulmonary embolism, and allergic reactions. Patients may also develop organ dysfunction that is related to disease progression rather than CRS. For these reasons, multidisciplinary management provides additional support in diagnosis. If there is concern for heart failure or pulmonary emboli, expert opinion from cardiology and pulmonology will prove invaluable. A list of differential diagnoses for CRS is presented in Table 2, along with clinical pearls to help differentiate from CRS. Table 3 provides a decision support tool for APPs.

**Table 2:** Differential diagnoses for cytokine release syndrome.

**Diagnosis**	**Clinical Pearls to Differentiate from CRS**
Infection/Sepsis	Assess for focal symptoms of infection; follow culture and imaging results; treat empirically for infection particularly in neutropenic patients. Often treated with anti-cytokine therapy simultaneously.
Tumor Lysis Syndrome	Monitor laboratory findings: elevated phosphate, uric acid, calcium, and potassium may help differentiate from CRS
Hypersensitivity	Occurs minutes to hour(s) after infusion; and more frequently in patients with >1 CAR T-cell infusion
Hemophagocytic lymphohistiocytosis (HLH)/macrophage activation syndrome (MAS)	If suspicious, refer to Histiocyte Society guidelines (33) which includes either molecular mutations OR five of the following: fever; splenomegaly; cytopenia affecting at least 2 different cell lines; hypertriglyceridemia; hemophagocytosis in bone marrow, lymph nodes, liver or spleen; low or absent natural killer cells; elevated ferritin; and increased soluble CD25 concentration (i.e., soluble IL-2 receptor)
Pulmonary Embolism	Evaluate by CT angiography; d-dimer often not helpful as it often elevated in patients with malignancy at baseline and coagulopathies can be related to CRS

*CRS, cytokine release syndrome; CAR T-cell, chimeric antigen receptor T-cell; CD25, clusters of differentiation 25, IL-2, interleukin 2; CT, computed tomography*.

**Table 3:** Decisional flowchart for cytokine release syndrome.

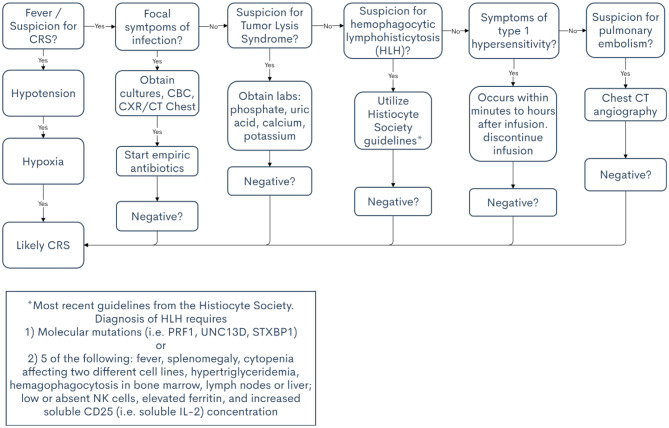

## Immune Effector Cell-Associated Neurotoxicity Syndrome (ICANS)

ICANS is the second most commonly noted adverse event related to CAR T-cell therapy. Immune effector cell-associated neurotoxicity syndrome has been defined by the ASTCT consensus group as “a disorder characterized by a pathologic process involving the central nervous system following any immune therapy that results in the activation or engagement of endogenous or infused T cells and/or other immune effector cells.” According to the ASTCT, symptoms of ICANS can include aphasia, altered level of consciousness, impaired cognitive skills, motor weakness, seizures, and cerebral edema, any of which may be progressive (17). Onset and severity of ICANS varies with therapeutic cell products used, but in general, median onset of ICANS in patient who have received commercially approved cell products is 4–6 days with a median duration of 1–14 days. Early manifestations of ICANS include tremor, mild aphasia, altered mental status with impaired attention and confusion, apraxia, and lethargy. These symptoms can progress until the patient becomes somnolent and/or obtunded. In severe cases, mechanical ventilation may be necessary (35). There have been numerous deaths from CAR T-cell therapy-related ICANS (12, 36, 37). Furthermore, one study showed that severe ICANS (grades 3 or 4) has been correlated with decreased overall survival (38). Patient and family education regarding CAR T-cell therapy-related neurotoxicity is paramount because these symptoms can be distressing to all involved.

### Grading of ICANS

In 2018, the ASTCT group developed the current consensus guidelines for the grading of immune effector cell-associated neurotoxicity syndrome (17). Encephalopathy is now graded by a tool termed the immune-effector cell-associated encephalopathy (ICE) score. The grading of ICANS includes the ICE score as well as depressed level of consciousness, seizure activity, motor findings, and raised ICP pressure/cerebral edema (Tables 4, 5). Correct attribution, i.e., directly relating adverse event to CAR T-cell therapy or not, is of high importance to avoid meaningfully affecting toxicity reporting and grading. For instance, it is common to attribute generalized weakness as focal motor weakness according to the ICANS grading system which could lead to unnecessary and costly testing. The remainder of this section outlines the risk factors and the differential diagnoses of ICANS to mitigate incorrect attribution.

**Table 4:** American Society for Transplantation and Cellular Therapy consensus encephalopathy assessment tool.

**Immune-effector cell-associated encephalopathy tool (ICE)**
• **Orientation:** Orientation to year, month, city, hospital: 4 points • **Naming:** Ability to name three objects (e.g., patient is asked to point to clock, pen, button): 3 points • **Following commands:** Ability to follow directions (e.g., patient is asked to hold up two fingers or close their eyes and stick out their tongue): 1 point • **Writing:** Ability to write a standard sentence (e.g., Our national bird is the bald eagle): 1 point • **Attention:** Ability to count backwards from 100 by 10: 1 point

*Table adapted from (17)*.

**Table 5:** American Society for transplantation and cellular therapy consensus grading of immune effector cell-associated neurotoxicity syndrome for adults.

**Neurotoxicity domain^**‡**^**	**Grade 1**	**Grade 2**	**Grade 3**	**Grade 4**
ICE score^∧^	7–9	3–6	0–2	0 (patient is unarousable and unable to be evaluated for ICE)
Depressed level of consciousness^*^	Awakens spontaneously	Awakens to voice	Awakens only to tactile stimulus	Patient is unarousable or requires vigorous or repetitive tactile stimuli to arouse or stupor or coma
Seizure	Not applicable	Not applicable	Any focal or generalized clinical seizures that resolves rapidly or non-convulsive seizures on EEG that resolve with intervention	Life-threatening prolonged seizures (>5 min); or Repetitive clinical to electrical seizures without return to baseline in between
Motor findings^æ^	Not applicable	Not applicable	Not applicable	Deep focal motor weakness such as hemiparesis or paraparesis
Raised intracranial pressure/cerebral edema	Not applicable	Not applicable	Focal/local edema detected by neuroimaging^#^	Diffuse cerebral edema detected by neuroimaging, decerebrate or decorticate posturing, or cranial nerve VI palsy, or papilledema; or Cushing triad

‡ *ICANS grade is determined by the most severe event (ICE score, level of consciousness, seizure, motor findings, and raised intracranial pressure/cerebral edema) not attributable to any other cause*.

∧ *A patient with an immune effector cell-associated encephalopathy (ICE) score of 0 may be classified as having grade 3 ICANS if the patient is awake with global aphasia. But a patient with an ICE score of 0 may be classified as having grade 4 ICANS if the patient is unarousable*.

* *Depressed level of consciousness should be attributable to no other cause (e.g., no sedating medication)*.

æ *Tremors and myoclonus associated with immune effector cell therapies may be graded according to Common Terminology Criteria for Adverse Events version 5.0 but they do not influence ICANS grading*.

# *Intra-cranial hemorrhage with or without associated edema is not considered a neurotoxicity feature and is excluded from ICANS grading. It may be graded according to Common Terminology Criteria for Adverse Events version 5.0*.

*Table adapted from (17)*.

### Risk Factors for ICANS

Factors that correlate with increased risk of ICANS include high tumor burden, lymphodepletion with cyclophosphamide and fludarabine, high CAR T-cell dose, and high ferritin and cytokine levels (39). Several studies have also shown that severe (often defined as grade 3 or 4) CRS is a predictive marker for the development of severe ICANS (12, 38–41). For instance, in numerous studies, 90% of patients who developed ICANS previously had CRS. Additionally, Karschnia et al. found that decreased platelets at the time of CAR T-cell infusion correlated with an increased grade of ICANS, possibly indicating that heavily pre-treated patients tend to have an increased risk of treatment-related adverse events (38). Furthermore, this finding may act as a future biomarker of blood-brain barrier disturbance which could suggest a pathogenesis of ICANS (38).

Although differentiating between ICANS and other neurologic conditions may be challenging, in one phase I study, expressive aphasia developed in 19 of 22 patients who eventually went on to develop severe ICANS (41). Thus, it is imperative that APPs have a thorough understanding of the neurological symptoms that may occur with ICANS in order to determine the correct underlying causes and treatment plan.

### Differential Diagnoses for ICANS

The differential diagnoses for ICANS most often involves infection and stroke. An infection accompanied by a high fever can cause confusion that presents similarly to ICANS, and this should be the first differential diagnosis to consider. Stroke risk is also increased in patients who have undergone CAR T-cell therapy owing to its potential to induce hypercoagulability (42). Patients may also be thrombocytopenic after lymphodepleting chemotherapy, potentially increasing the risk for cerebral bleed (43). An understanding of stroke presentation and diagnosis is necessary when treating patients who have received CAR T-cell therapy because the symptoms of stroke and ICANS can be strikingly similar. Other causes of confusion besides ICANS may include sepsis, pain medications, and disease progression in the central nervous system, such as leptomeningeal disease or parenchymal brain lesions. A list of differential diagnoses, including clinical pearls associated with each, are included in Table 6. Additionally, a decisional flowchart for APPs in provided in Table 7.

**Table 6:** Differential diagnoses for immune effector cell-associated neurotoxicity syndrome.

**Diagnosis**	**Clinical pearls to differentiate from ICANS**
Infection/Sepsis	High fever can lead to confusion; obtain cultures; and labs
Stroke	Higher risk in these patients; may require initial non-contrast CT of head
Pain medication	Review medication administration record for past 24–72 h
New or progressive disease in the central nervous system	Leptomeningeal disease and/or parenchymal brain lesions; found on imaging and/or lumbar puncture cytology

**Table 7:** Decisional flowchart for immune effector cell-associated neurotoxicity syndrome.

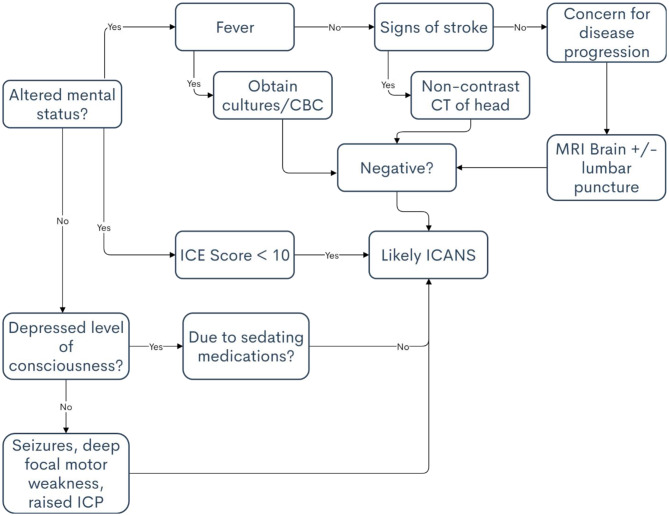

*ICE, immune-effector cell-associated encephalopathy tool; ICP, intracranial pressure detected by neuroimaging*.

Consideration of the timeframe in which the symptoms present is helpful in determining the diagnosis. It is rare, among the current commercial products, for a patient to develop ICANS >30 days after infusion, and even more rare past 60 days. If neurological symptoms present past this time-frame include the aforementioned diagnoses ahead of ICANS when formulating a differential diagnosis. The time-frame and incidence of ICANS does vary to an extent among products, so a familiarity with the product is helpful in differentiating among neurological conditions. Additionally, multidisciplinary management can aide in diagnosis. As described, encephalopathy can manifest in a myriad of ways, so a consult with neurology is often required. Should mechanical ventilation be necessary pulmonary and respiratory therapy must also be skilled in proper diagnosis and timely intervention. Infectious disease experts, while often called upon during CRS, are often required when there is suspicion for ICANS.

## Other Adverse Events

### B-Cell Aplasia/Cytopenias/ Hypogammaglobulinemia

Although CD19 is expressed on most B-cell malignancies, it is also present on normal B-cells, creating an “on-target, off-tumor” effect. This is termed B-cell aplasia, which results in cytopenias and hypogammaglobulinemia. Cytopenias typically occurs within the first 30 days after cell infusion but can take months or possibly longer to resolve (44). Lymphodepleting chemotherapies are known to cause drops in blood counts initially, but the CAR T-cells can also cause immune-mediated pancytopenia. Thus, it is important to monitor blood counts closely until they return to normal and to provide support with transfusions and growth factors as indicated. However, with some cell products, growth factors should be avoided for a certain period after cell infusion.

According to a number of studies, infection incidence and severity decreased after day 30 of cell infusion (29). Still, given the potential for prolonged B-cell aplasia and subsequent infections, prophylaxis should be considered. At our institution, we recommend herpes zoster and *Pneumocystis jiroveci* prophylaxis for at least 1 year post CAR T-cell therapy. After 1 year, *Pneumocystis jiroveci* prophylaxis is discontinued if CD4 counts are >200 and viral prophylaxis is discontinued upon CD19 recovery. Antibacterial and antifungal prophylaxis is initiated at the time of infusion and continued until the absolute neutrophil count is >0.5 K/μL for 3 consecutive days without growth factor support. For patients who develop frequent viral infections and have low levels of immunoglobulin G, monthly infusions of intravenous immunoglobulin G or weekly administration of the subcutaneous formulation is recommended.

It is also important to consider vaccinations in patients who have undergone CAR T-cell therapy. If the patient has undergone stem cell transplantation previously and was never vaccinated after the transplantation, our institution follows the autologous stem cell transplantation and allogeneic stem cell transplantation guidelines from the Centers for Disease Control and Prevention (10). It may benefit patients to wait until their B-cells have recovered or antibodies may not be formed. However, seasonal influenza may be the exception because T-cell response has been reported (45).

## Multidisciplinary Team Management

As previously highlighted, a multidisciplinary approach is required to effectively manage the toxicities associated with CAR T-cell therapy. Because CRS may lead to multiple organ system failure, experts in cardiology, nephrology, and pulmonology are often consulted (46). It is no uncommon for a patient to experience an acute kidney injury or re-activation of hepatitis which requires consult with colleagues in nephrology and hepatology. Infectious disease specialists assist with differentiating infection from CRS and offer advice on the treatment of rare infections (29, 42). The neurology team provides expertise in assessing and managing ICANS including seizure management. Psychiatry specialists may also be involved in assisting with neuropsychiatric effects (11). Intensivists frequently care for patients who develop grade 3 and 4 toxicities (47). Emergency room physicians encounter patients who return to the hospital with complications after CAR T-cell therapy. Pharmacists play a role in medication management which assists in ruling out other medications as causes for symptoms as well as vaccination in post CAR T-cell recipients (15, 48). In both inpatient and outpatient settings, nurses play a pivotal role in identifying toxicities and informing the medical team in a timely manner so that appropriate interventions can be initiated (49). APPs are often members of both the primary and consulting teams and follow patients throughout the continuum of care in both inpatient and outpatient settings, and APPs are frequently the frontline personnel in providing rapid assessment of toxicities and/or following the long-term and late effects of CAR T-cell therapy (50).

## Challenges and Opportunities for APPs

As additional CAR T-cell therapies receive FDA approval for commercial use and new targets and constructs are developed, including for use in solid tumors, more centers will be providing CAR T-cell therapy (51). APPs who practice oncology will experience both the challenges and opportunities that occur with exponentially increased use of this therapy.

Many of the current CAR T-cell products are known to cause toxicities. Approximately 30% of patients develop high-grade CRS or ICANS with the currently available products. APPs are in a position to effectively provide high-level rapid assessment of toxicities and foster early intervention through initiation of treatment algorithms and communication with nurses and physicians.

Currently, there are challenges regarding consistent grading of toxicities, which is important because the grade of the toxicity determines the treatment. The ASTCT guidelines for immune effector cell toxicities have provided a foundation for consensus; however, there are currently active clinical trials for CAR T-cell therapy that still use the Lee and other criteria for grading. This leads to confusion and difficulty in comparing results across trials or products. This will likely resolve over time as new clinical trials incorporate the ASTCT guidelines and institutions adopt the ASTCT consensus grading. Knowledgeable APPs also have the opportunity to re-inforce education about grading for nurses and fellow APPs.

Many clinicians interact with patients receiving CAR T-cell therapy. All physicians, nurses, APPs, and pharmacists involved in caring for patients receiving CAR T-cell therapy are required to complete product-specific Risk Evaluation and Mitigation Strategy training for commercially available immune effects cell therapies. The Foundation for the Accreditation of Cellular Therapy has also set standards for clinical practice, including requirements for continuing education regarding immune effector cell therapy. Ensuring that all members of the team remain competent in caring for patients receiving these therapies may be a challenge initially, but this goal becomes more feasible as experience grows. APPs have the opportunity to become experts in this new therapy and to educate colleagues through speaking engagements and publications.

## Conclusion

Immunotherapy is rapidly becoming a cornerstone of treatment for cancer. The number of patients receiving immune effector cell therapy such as CAR T-cell therapy continues to grow as new targets and constructs are developed. As members of a multi-disciplinary team caring for these patients, APPs have the opportunity to improve outcomes for this patient population by developing expertise in the recognition, grading and treatment of acute toxicities and the management of potential long-term and late effects of this therapy.

## Author Contributions

SS was responsible for the development and writing of the manuscript. GW, SJ, and SA contributed equally to the review and revision of the manuscript. All authors contributed to the article and approved the submitted version.

## Conflict of Interest

SA has participated on Advisory Boards for Kite/Gilead and Celgene. The remaining authors declare that the research was conducted in the absence of any commercial or financial relationships that could be construed as a potential conflict of interest.

## References

[1] Yu S Yi M Qin S Wu K. Next generation chimeric antigen receptor T cells: safety strategies to overcome toxicity. Mol Cancer. (2019) 18:125. 10.1186/s12943-019-1057-4 31429760 PMC6701025

[2] Coscia M Vitale C Cerrano M Maffini E Giaccone L Boccadoro M. Adoptive immunotherapy with CAR modified T cells in cancer: current landscape and future perspectives. Front Biosci. (2019) 24:1284–315. 10.2741/4780 31136980

[3] Strati P Neelapu SS. Chimeric antigen receptor-engineered T cell therapy in lymphoma. Curr Oncol Rep. (2019) 21:38. 10.1007/s11912-019-0789-z 30919158

[4] Tang XY Sun Y Zhang A Hu GL Cao W Wang D-H. Third-generation CD28/4-1BB chimeric antigen receptor T cells for chemotherapy relapsed or refractory acute lymphoblastic leukaemia: a non-randomised, open-label phase I trial protocol. BMJ Open. (2016) 6:e013904. 10.1136/bmjopen-2016-013904 28039295 PMC5223707

[5] Neelapu SS Locke FL Bartlett NL Lekakis LJ Miklos DB Jacobson CA. Axicabtagene ciloleucel CAR T-cell therapy in refractory large B-cell lymphoma. N Engl J Med. (2017) 377:2531−44. 10.1056/NEJMoa1707447 29226797 PMC5882485

[6] Schuster SJ Bishop MR Tam CS Waller EK Borchmann P McGuirk JP Tisagenlecleucel in adult relapsed or refractory diffuse large B-cell lymphoma. N Engl J Med. (2019) 380:45–56. 10.1056/NEJMoa1804980 30501490

[7] Chavez JC Bachmeier C Kharfan-Dabaja MA CAR T-cell therapy for B-cell lymphomas: clinical trial results of available products. Ther Adv Hematol. (2019) 10:2040620719841581 10.1177/2040620719841581 31019670 PMC6466472

[8] Maude SL Teachey DT Rheingold SR Shaw PA Aplenc R Barrett DM. Sustained remissions with CD19-specific chimeric antigen receptor (CAR)-modified T cells in children with relapsed/refractory ALL. Journal of Clinical Oncology. (2016) 34(15_Suppl):3011. 10.1200/JCO.2016.34.15_suppl.301125696911

[9] Jacoby E Shahani SA Shah NN. Updates on CAR T-cell therapy in B-cell malignancies. Immunol Rev. (2019) 290:39–59. 10.1111/imr.12774 31355492

[10] Jain T Bar M Kansagra AJ Chong EA Hashni SK Neelapu SS. Use of chimeric antigen receptor T cell therapy in clinical practice for relapsed/refractory aggressive B cell non-hodgkin lymphoma: an expert panel opinion from the American Society for transplantation and cellular therapy. Biol Blood Marrow Transplant. (2019) 25:2305–21. 10.1016/j.bbmt.2019.08.015 31446199

[11] Ruark J Mullane E Cleary N Cordeiro A Bezerra ED Wu V. Patient-reported neuropsychiatric outcomes of long-term survivors after chimeric antigen receptor T cell therapy. Biol Blood Marrow Transplant. (2019) 26:34–43. 10.1016/j.bbmt.2019.09.037 31605820 PMC6951812

[12] Neelapu SS Tummala S Kebriaei P Wierda W Gutierrez C Locke FL. Chimeric antigen receptor T-cell therapy - assessment and management of toxicities. Nat Rev Clin Oncol. (2018) 15:47–62. 10.1038/nrclinonc.2017.148 28925994 PMC6733403

[13] Giavridis T van der Stegen SJC Eyquem J Hamieh M Piersigilli A Sadelain M. CAR T cell-induced cytokine release syndrome is mediated by macrophages and abated by IL-1 blockade. Nat Med. (2018) 24:731–8. 10.1038/s41591-018-0041-7 29808005 PMC6410714

[14] Taraseviciute A Tkachev V Ponce R Turtle CJ Snyder JM Liggitt D. Chimeric antigen receptor T cell-mediated neurotoxicity in nonhuman primates. Cancer Discov. (2018) 8:750–63. 10.1158/2159-8290.CD-17-1368 29563103 PMC6058704

[15] Dushenkov A Jungsuwadee P. Chimeric antigen receptor T-cell therapy: foundational science and clinical knowledge for pharmacy practice. J Oncol Pharm Pract. (2019) 25:1217–25. 10.1177/1078155219836480 30890066

[16] Heslop HE Shpall EJ. Harmonizing immune effector toxicity reporting. Biol Blood Marrow Transplant. (2019) 25:e121–2. 10.1016/j.bbmt.2019.01.001 30615980

[17] Lee DW Santomasso BD Locke FL Ghobadi A Turtle CJ Brudno JN. ASTCT consensus grading for cytokine release syndrome and neurologic toxicity associated with immune effector cells. Biol Blood Marrow Transpl. (2019) 25:625–38. 10.1016/j.bbmt.2018.12.758 30592986 PMC12180426

[18] Kansagra AJ Frey NV Bar M Laetsch TW Carpenter PA Savani BN Clinical utilization of chimeric antigen receptor T cells in B cell acute lymphoblastic leukemia: an expert opinion from the European Society for blood and marrow transplantation and the American Society for blood and marrow transplantation. Biol Blood Marrow Transplant. (2019) 25:e76–85. 10.1016/j.bbmt.2018.12.068 30576834 PMC8335749

[19] Frey N Porter D. Cytokine release syndrome with chimeric antigen receptor T cell therapy. Biol Blood Marrow Transplant. (2019) 25:e123–7. 10.1016/j.bbmt.2018.12.756 30586620

[20] Adkins S The role of advanced practitioners in optimizing clinical management and support of patient with cytokine release syndrome from CAR T-cell therapy. J Adv Pract Oncol. (2019) 10:830–43. 10.6004/jadpro.2019.10.8.5 PMC7517761 33425467

[21] Park JH Riviere I Gonen M Wang X Senechal B Curran KJ. Long-term follow-up of CD19 CAR therapy in acute lymphoblastic leukemia. N Engl J Med. (2018) 378:449–59. 10.1056/NEJMoa1709919 29385376 PMC6637939

[22] Porter D Frey N Wood PA Weng Y Grupp SA Grading of cytokine release syndrome associated with the CAR T cell therapy tisagenlecleucel. J Hematol Oncol. (2018) 11:35 10.1186/s13045-018-0571-y 29499750 PMC5833070

[23] Lee DW Gardner R Porter DL Louis CU Ahmed N Jensen M. Current concepts in the diagnosis and management of cytokine release syndrome. Blood. (2014) 124:188–95. 10.1182/blood-2014-05-552729 24876563 PMC4093680

[24] Shimabukuro-Vornhagen A Godel P Subklewe M Stemmler HJ Schlober HA Schlaak M. Cytokine release syndrome. J Immunother Cancer. (2018) 6:56. 10.1186/s40425-018-0343-9 29907163 PMC6003181

[25] Turtle CJ Hanafi L-A Berger C Gooley TA Cherian S Hudecek M. CD19 CAR?T cells of defined CD4+:CD8+ composition in adult B cell ALL patients. J Clin Investig. (2016) 126:2123. 10.1172/JCI85309 27111235 PMC4887159

[26] Ruella M Kenderian SS Shestova O Klichinsky M Melenhorst JS Wasik MA. Kinase inhibitor ibrutinib to prevent cytokine-release syndrome after anti-CD19 chimeric antigen receptor T cells for B-cell neoplasms. Leukemia. (2017) 31:246–8. 10.1038/leu.2016.262 27677739

[27] Teachey DT Lacey SF Shaw PA Melenhorst J Maude SL Frey N. Identification of predictive biomarkers for cytokine release syndrome after chimeric antigen receptor T-cell therapy for acute lymphoblastic leukemia. Cancer Discov. (2016) 6:664–79. 10.1158/2159-8290.CD-16-0040 27076371 PMC5448406

[28] Brentjens RJ Davila ML Riviere I Park J Wang X Cowell CG. CD19-targeted T cells rapidly induce molecular remissions in adults with chemotherapy-refractory acute lymphoblastic leukemia. Sci Transl Med. (2013) 5:177ra138. 10.1126/scitranslmed.3005930 23515080 PMC3742551

[29] Hill JA Li D Hay KA Green ML Cherian S Chen X. Infectious complications of CD19-targeted chimeric antigen receptor-modified T-cell immunotherapy. Blood. (2018) 131:121–30. 10.1182/blood-2017-07-793760 29038338 PMC5755046

[30] Singer M Deutschman CS Seymour CW Shankar-Hari M Annane D Bauer M The third international consensus definitions for sepsis and septic shock (Sepsis-3). JAMA. (2016) 315:801–10. 10.1001/jama.2016.0287 26903338 PMC4968574

[31] Seymour CW Liu VX Iwashyna TJ Brunkhorst FM Rea TD Scherag A Assessment of clinical criteria for sepsis: for the third international consensus definitions for sepsis and septic shock (Sepsis-3). JAMA. (2016) 315:762–74. 10.1001/jama.2016.0288 26903335 PMC5433435

[32] Maus MV Haas AR Beatty GL Albelda SM Levine BL Lin X T cells expressing chimeric antigen receptors can cause anaphylaxis in humans. Cancer Immunol Res. (2013) 1:26–31. 10.1158/2326-6066.CIR-13-0006 PMC3888798 24777247

[33] Ramos-Casals M Brito-Zeron P Lopez-Guillermo A Khamashta MA Bosch X. Adult haemophagocytic syndrome. Lancet. (2014) 383:1503–16. 10.1016/S0140-6736(13)61048-X 24290661

[34] Hayden A Park S Giustini D Lee AY Chen LY. Hemophagocytic syndromes (HPSs) including hemophagocytic lymphohistiocytosis (HLH) in adults: a systematic scoping review. Blood Rev. (2016) 30:411–20. 10.1016/j.blre.2016.05.001 27238576

[35] Hunter BD Jacobson CA CAR T-cell associated neurotoxicity: mechanisms, clinicopathologic correlates, and future directions. J Natl Cancer Inst. (2019) 111:646–54. 10.1093/jnci/djz017 30753567

[36] Johnson LA June CH. Driving gene-engineered T cell immunotherapy of cancer. Cell Res. (2017) 27:38–58. 10.1038/cr.2016.154 28025979 PMC5223234

[37] Turtle CJ Hay KA Hanafi LA Li D Myerson D Gonzalez-Cuyar LF. Durable molecular remissions in chronic lymphocytic leukemia treated with CD19-specific chimeric antigen receptor-modified T cells after failure of ibrutinib. J Clin Oncol. (2017) 35:3010–20. 10.1200/JCO.2017.72.8519 28715249 PMC5590803

[38] Karschnia P Jordan JT Forst DA Arrillaya-Romany IL Batchelor TT Baehringet JM. Clinical presentation, management, and biomarkers of neurotoxicity after adoptive immunotherapy with CAR T cells. Blood. (2019) 133:2212–21. 10.1182/blood-2018-12-893396 30808634

[39] Gust J Hay KA Hanafi LA Li D Cherian S Chen X. Endothelial activation and blood-brain barrier disruption in neurotoxicity after adoptive immunotherapy with CD19 CAR-T cells. Cancer Discov. (2017) 7:1404–19. 10.1158/2159-8290.CD-17-0698 29025771 PMC5718945

[40] Schuster SJ Svoboda J Chong EA Nasta SD Mato AR Anak O. Chimeric antigen receptor T cells in refractory B-cell lymphomas. N Engl J Med. (2017) 377:2545–54. 10.1056/NEJMoa1708566 29226764 PMC5788566

[41] Santomasso BD Park JH Salloum D Riviere I Flynn J Mead E. Clinical and biological correlates of neurotoxicity associated with CAR T-cell therapy in patients with B-cell acute lymphoblastic leukemia. Cancer Discov. (2018) 8:958–71. 10.1158/2159-8290.CD-17-1319 29880584 PMC6385599

[42] Jiang H Liu L Guo T Wu Y Ai L Deng J. Improving the safety of CAR-T cell therapy by controlling CRS-related coagulopathy. Ann Hematol. (2019) 98:1721–32. 10.1007/s00277-019-03685-z 31055613

[43] Hay KA Hanafi LA Li D Gust J Liles WC Wurfel MM. Kinetics and biomarkers of severe cytokine release syndrome after CD19 chimeric antigen receptor-modified T-cell therapy. Blood. (2017) 130:2295–306. 10.1182/blood-2017-06-793141 28924019 PMC5701525

[44] Strati P Adkins S Nastoupil LJ Westin J Hagemeister FB Fowler NH Hematopoietic recovery and immune reconstitution after axi-cel CAR T-cell therapy in patients with relapsed/refractory large B-cell lymphoma. J Clin Oncol. (2019) 37(15_suppl):7545 10.1200/JCO.2019.37.15_suppl.7545

[45] Ljungman P Avetisyan G. Influenza vaccination in hematopoietic SCT recipients. Bone Marrow Transplant. (2008) 42:637–41. 10.1038/bmt.2008.264 18724396

[46] Rashdan S Minna JD Gerber DE. Diagnosis and management of pulmonary toxicity associated with cancer immunotherapy. Lancet Respir Med. (2018) 6:472–8. 10.1016/S2213-2600(18)30172-3 29856320 PMC7341891

[47] Azoulay E Shimabukuro-Vornhagen A Darmon M von Bergwelt-Baildon M. Critical care management of chimeric antigen receptor T cell-related toxicity. Be aware and prepared. Am J Respir Crit Care Med. (2019) 200:20–3. 10.1164/rccm.201810-1945ED 30676776

[48] Mahmoudjafari Z Hawks KG Hsieh AA Plesca D Gatwood KS Culos KA. American Society for Blood and Marrow Transplantation Pharmacy Special Interest Group Survey on chimeric antigen receptor T cell therapy administrative, logistic, and toxicity management practices in the United States. Biol Blood Marrow Transplant. (2019) 25:26–33. 10.1016/j.bbmt.2018.09.024 30266675

[49] Halton E Llerandi D Diamonte C Quintanilla H Miale-Mayer D. Developing infrastructure: managing patients with cancer undergoing CAR T-cell therapy. Clin J Oncol Nurs. (2017) 21(2 Suppl):35–40. 10.1188/17.CJON.2.35-40 28315556

[50] Adkins S CAR T-cell therapy: adverse events and management. J. Adv. Pract. Oncol. (2019) 10(Suppl 3):21–38. 10.6004/jadpro.2019.10.4.11 PMC7521123 33520343

[51] Liu E Marin D Banerjee P Macapinlac HA Thompson P Basar R. Use of CAR-transduced natural killer cells in CD19-positive lymphoid tumors. N. Engl. J. Med. (2020) 382:545–53. 10.1056/NEJMoa1910607 32023374 PMC7101242

